# Regeneration of a neoartery through a completely autologous acellular conduit in a minipig model: a pilot study

**DOI:** 10.1186/s12967-018-1763-5

**Published:** 2019-01-11

**Authors:** Tao Wang, Nianguo Dong, Huimin Yan, Sze Yue Wong, Wen Zhao, Kang Xu, Dong Wang, Song Li, Xuefeng Qiu

**Affiliations:** 1 Department of Cardiovascular Surgery, Union Hospital, Tongji Medical College, Huazhong University of Science and Technology, 1277 Jiefang Ave, Wuhan, 430022 China; 2 Department of Bioengineering, University of California, Berkeley, CA 94720 USA; 3 Bioprocessing Technology Institute, Agency for Science, Technology and Research (A*STAR), Singapore, 138668 Singapore; 4 Key Laboratory for Space Biosciences and Biotechnology, School of Life Sciences, Northwestern Polytechnical University, Xi’an, 710072 Shaanxi China; 5 Department of Bioengineering, University of California, Los Angeles, CA 90095 USA; 6 Department of Medicine, University of California, Los Angeles, Los Angeles, CA 90095 USA; 7 Present Address: Department of Thoracic and Cardiovascular Surgery, Central Hospital of Zhuzhou, Zhuzhou, 412000 Hunan China

**Keywords:** Vascular graft, Extracellular matrix, Autologous graft, Remodeling

## Abstract

**Background:**

Vascular grafts are widely used as a treatment in coronary artery bypass surgery, hemodialysis, peripheral arterial bypass and congenital heart disease. Various types of synthetic and natural materials were experimented to produce tissue engineering vascular grafts. In this study, we investigated in vivo tissue engineering technology in miniature pigs to prepare decellularized autologous extracellular matrix-based grafts that could be used as vascular grafts for small-diameter vascular bypass surgery.

**Methods:**

Autologous tissue conduits (3.9 mm in diameter) were fabricated by embedding Teflon tubings in the subcutaneous pocket of female miniature pigs (n = 8, body weight 25–30 kg) for 4 weeks. They were then decellularized by CHAPS decellularization solution. Heparin was covalently-linked to decellularized tissue conduits by Sulfo-NHS/EDC. We implanted these decellularized, completely autologous extracellular matrix-based grafts into the carotid arteries of miniature pigs, then sacrificed the pigs at 1 or 2 months after implantation and evaluated the patency rate and explants histologically.

**Results:**

After 1 month, the patency rate was 100% (5/5) while the inner diameter of the grafts was 3.43 ± 0.05 mm (n = 5). After 2 months, the patency rate was 67% (2/3) while the inner diameter of the grafts was 2.32 ± 0.14 mm (n = 3). Histological staining confirmed successful cell infiltration, and collagen and elastin deposition in 2-month samples. A monolayer of endothelial cells was observed along the inner lumen while smooth muscle cells were dominant in the graft wall.

**Conclusion:**

A completely autologous acellular conduit with excellent performance in mechanical properties can be remodeled into a neoartery in a minipig model. This proof-of-concept study in the large animal model is very encouraging and indicates that this is a highly feasible idea worthy of further study in non-human primates before clinical translation.

## Background

Ischaemic heart disease became the leading cause of death around the world, vascular bypass surgery remains the primary treatment for patients with ischaemic heart disease [1], as well as being widely used in lower extremity bypass [2]. Autologous vessels such as saphenous veins and internal mammary arteries are the gold-standard grafts used as vascular prosthesis. Unfortunately, these vessels are not always available or suitable for surgical procedure due to former bypass surgery or pre-existing vascular disease [3–5].

When autologous vessels are not available, other arti Figure cial grafts are often used. Dacron and polytetrafluoroethylene (PTFE) are two synthetic materials that are widely used for large-diameter (≥ 6 mm) grafts, such as arteriovenous access for hemodialysis, peripheral arterial bypass above the knee [6]. Unfortunately, these synthetic materials have failed when used for small-diameter (< 6 mm) grafts due to infection, thrombus formation or intimal hyperplasia [7]. Over the past three decades, tissue engineering vascular grafts (TEVGs) were widely studied to explore grafts which were immunologically compatible, nonthrombogenic and can grow and be remolded. Numerous TEVGs such as cell seeded synthetic grafts, collagen and fibrin-based vessels, cell self-assembly vessels and biodegradable synthetic polymer scaffolds have been developed. However, immune response, thrombus formation, intimal hyperplasia, low mechanical strength and a complicated processing procedure limit their clinical applications [8–12].

A novel method to fabricate TEVGs utilizing in vivo tissue engineering technology was first proposed by Sparks et al. [13]. Briefly, a rod was inserted under the skin and, over a period of time, the granulation tissue was formed. The rod was then removed leaving the autologous tissue conduit which can be used as a vascular graft. This kind of grafts can be easily obtained and were immune-free to the host, but they failed due to poor burst strength and low patency rate. Following this, both Campbell and Nakayama proposed modifications to the process. Campbell et al. employed a silastic tube as a rod to be inserted and everted the tissue capsule to ensure the inner wall was covered with non-thrombotic mesothelial cells [14]. Meanwhile, Nakayama et al. adopted silicone rods and coated the tissue capsule with argatroban [15]. Although advances were made, the complications and unsatisfactory patency rate caused by relatively poor mechanical properties, thrombus formation and intimal hyperplasia make them inappropriate for clinical use. Niklason et al. and Tedd et al. engineered the decellularized extracellular matrix vascular grafts using human allogeneic or autologous cells for chronic haemodialysis. Therefore, this research seeks to describe the methods to develop decellularized completely autologous extracellular matrix-based grafts with appropriate mechanical properties and anticoagulation properties. These grafts are easily prepared, immunologically compatible and appropriate for cell migration and growth and can be remolded into the neoartery in a minipig model.

## Materials and methods

### Preparation of autologous tissue conduits

The Teflon tubings (out diameter 3.9 mm) were tailored into 6 cm long segments, then immersed in 75% alcohol for sterilization. Miniature pigs (n = 8, female) weighing 25–30 kg were injected intramuscularly with zolazepam (5 mg/kg), xylazine (2.25 mg/kg) and atropine (1 mg) for pre-anesthesia, the pigs were masked with isoflurane to induce sedation, endotracheal tube was positioned through the mouth, ventilate the animal with a mixture of oxygen and nitrogen (1:2 v/v), using the following ventilator settings: Pressure control mode: positive end-expiratory pressure (PEEP) 4 cm H_2_O; peak inspiratory pressure 16–18 cm H_2_O; breathing frequency 12–16 times per minute; this should result in a tidal volume of ~ 10 ml/kg. general anesthesia was induced by means of intravenous administration of fentanyl (10 μg/kg/h) via the ear vein catheter.

After well anesthesia, pigs were placed at the animal experimental platform with dorsal position, the abdominal skin was prepared without hair, disinfected three times with 0.1% povidone iodine. A small horizontal incision about 3 cm was made by surgical knife, then a longitudinal subcutaneous pocket was formed by blunt dissection and the tubings were inserted in this pocket, and a total of 8 tubings were inserted through 4 small incisions for each pig. Pigs were then send back to cages with conventional feeding. After 4 weeks, tubings with tissue conduits formed around them were harvested, extracted the tubings away and cleaned of excess tissue, left the tissue conduits, then stored them in 4 °C PBS with 1% penicillin and streptomycin for further proceeding. The pigs were kept alive for autologous implantation. All pigs received humane care and treatment in accordance with the ‘‘Guide for the Care and Use of Laboratory Animals in Huazhong University of Science and Technology’’.

### Decellularization of autologous tissue conduits

The prepared tissue conduits were washed with PBS for 3 times, then they were decellularized by incubating them in a solution of 8 mM CHAPS, 1 M NaCl, 0.12 mM NaOH and 25 mM EDTA in d H_2_O for 2 h on a stir plate at 37 °C, repeated for 5 times with refreshed decellularization solution. After 5 circles, the grafts were then washed 3 times in PBS for 10 min on a stir plate. After the decellularization process, grafts were stored in 4 °C PBS with 1% penicillin and streptomycin for further proceeding or testing.

### DNA quantification

The total amount of DNA of non-decellalarized or decellularized grafts was quantified using a DNA isolation kit for tissues. Briely, 40 mg wet weight samples were digested using a cell lysis buffer and Proteinase K followed by a protein precipitation solution and centrifugation to remove the protein fraction. In order to isolate the DNA, the supernatant was added with isopentanol and ethanol and then centrifuged and rehydrated with a DNA rehydrating solution. Total DNA was finally quantified using a spectrophotometer at 260 nm.

### Mechanical properties

#### Burst pressure

Burst pressure were measured by a customer designed flow system with the pressure transducer. 5 cm long grafts were attached to the flow system, PBS was injected into the flow system, and the pressure was increased in 50 mmHg increments until the graft failed, generally by pinhole leak or rupture. Record the maximum pressure as the burst pressure.

#### Suture retention strength

This test is intended to determine the force necessary to pull a suture from the prosthesis or cause the wall of the prosthesis to fail. Suture strength was measured in accordance with the ANSI/AAMI/ISO 7198 standard. A 2 cm long graft was prepared, three 6-0 prolene sutures, separated by 120°, were inserted 2 mm from the end of the graft through one wall to form a half loop. The sutures were pulled at the rate of 50 mm/min using an SANS Electromechanical Universal Testing Machine (IS 09001, SHENZHEN SANS TESTING MACHINE CO. LED), the three results were averaged for each sample.

#### Uniaxial traction testing

Grafts were cut into 5 mm long rings, measure and record the thickness and length of the rings in millimeters. Thread the specimen over the two pins subjected to SANS Electromechanical Universal Testing Machine. Samples were pre-loaded to 10% of failure strain prior to testing, stretch the specimen at a steady rate of 50 mm/min until the break point was reached. Ultimate tensile strength (UTS) and ultimate strain were defined by the peak stress and maximum deformation withstood by the samples prior to failure.

### Covalently linked with heparin

Heparin was covalently linked to decellularized tissue conduits by the crosslinks between succinimidyl esters on the heparin and amino functions on the collagen, while the succinimidyl esters was activated from Carboxylic acid groups of heparin (Hep-COOH) by EDC and NHS. More detailly, heparinzation solution was consisted of solution A and solution B, where solution A was prepared with EDC (40 mg/ml) and Sulfo-NHS (20 mg/ml) in MES buffer (0.05 M, PH = 5.5) over 12 h at room temperature, solution B was prepared with heparin sodium (60 mg/ml) in MES buffer (0.05 M, PH = 5.5). Fresh heparinization solution was prepared by mixing solution A and solution B together with volume ratio 1:1 at room temperature for 30 min, PH was adjusted to 7 with 1 M NaOH solution, then sterilized with 2 μm filter. Decellularized grafts were immersed in freshly prepared heparinization solution on a stir plate at room temperature for 5 h. After the heparinization procedure, grafts were washed with PBS.

The heparin-linked grafts were qualitative observed by toluidine blue staining. Briefly, 0.0005% toluidine blue solution was prepared in 0.01 M hydrochloric acid with 0.2% (w/v) sodium chloride, heparin-linked grafts were incubated in the toluidine blue solution overnight, heparin free grafts incubated as control. The blue-purple color appeared on the grafts indicated that heparin was linked to grafts.

The heparin-linked grafts were tailored into 5 mm long segments, weighed them and dissolved them in 5 ml chloroform/acetone (1:1 v/v ratio), the solution was centrifuged at 14,000*g* for 10 min, keep the precipitate, wash the precipitate with chloroform/acetone solution for 2 times, the organic solvent was evaporated at room temperature, leaving the heparin pellet extracted from the grafts. Next, immersed them in 8 ml toluidine blue/n-hexane (1:1 v/v ratio) solution for 6 h, meanwhile, a series of standard heparin with known concentration was incubated in 8 ml toluidine blue/n-hexane (1:1 v/v ratio) solution for 6 h also. The absorbance of both samples and standard heparin solution were measured at 630 nm using a plate reader.

The heparin-linked grafts were incubated in PBS, shaken in a water bath at 37 °C for 12 h, then for qualitative test as introduced above.

### Implantation and explantation in porcine models

From 3 days before surgery, aspirin (5 mg/kg) and clopidogrel (1 mg/kg) were given with one time each day. Aspirin and clopidogrel were continued daily till grafts explantation. After anesthetized by former methods, pigs were placed at the animal experimental platform with dorsal position, mark one 10 cm line above the left common carotid artery (1.5 cm apart from median line). The ear margin venous channels were established to insure liquid injection. Strict aseptic conditions and techniques were used for all procedures. The skin of the incision was cutted by surgical knife along the mark line, subcutaneous tissues were carefully dissected with an electric knife, superficial muscles were separated by blunt dissection, then cleidomastoid was well exposed. Along the gap between cleidomastoid and trachea, find the carotid sheath, the left common carotid artery was carefully dissected from the surrounding tissues by blunt dissection. Before clamping the artery, heparin (100 IU/kg) was administered intravenously, the artery was rinsed by glyceryl trinitrate to avoid angiospasm. The left common carotid artery was clamped with atraumatic vascular clamps (5.7 cm), approximate 5 cm long artery between the two clamps was dissected, tissue conduits (decellularized and heparinized) were implanted using 6-0 prolene as an end-to-end anastomosis to the common carotid artery, by interrupted suturation.. The animals were randomly divided into two groups, and the vascular prostheses were retrieved when the animals were killed at 1 (n = 5) and 2 (n = 3) months after implantation. The patency was inspected by duplex ultrasound before explantation. Heparin (100 IU/kg) was administered intravenously, clamp the proximal and distal artery, the implanted grafts were removed from the left common carotid artery. The removed grafts were cross-segmented and washed with PBS, then fixed with formalin or stored at − 80 °C for further test.

### Histological analysis

The native common carotid arteries, decellularized tissue conduits and post-implantation grafts were fixed in formalin and paraffin-embedded, 5 μm thick sections were cut using a microtome. Hematoxylin–eosin, Masson’s trichrome, Verhoeff staining were employed for histological analysis. To identify endothelial cells and smooth muscle cells, immunostaining was performed with antibody to Von Willebrand factor (abcam, ab6994) and alpha smooth muscle actin (abcam, ab7817).

### Statistical analysis

The results of quantitative studies were expressed as the mean ± standard deviation, a standard t test was used to determine the significance of the difference between the 2 means, and analyzed using a statistical analysis software IMB SPSS Statistics 19. p < 0.05 was considered statistically significant.

## Results

### Preparation of autologous tissue conduits

**Fig. 1:**
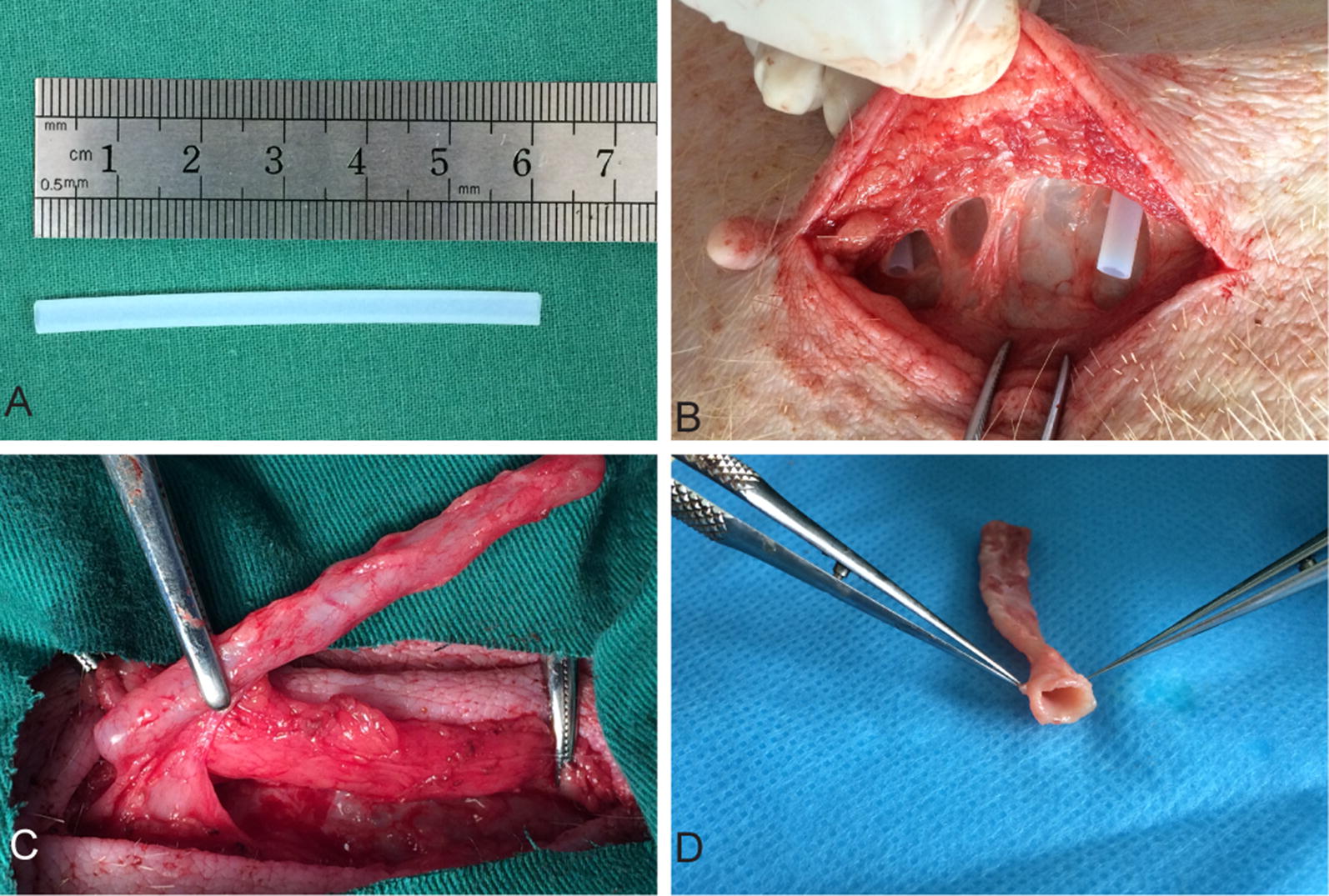
Preparation of an autologous tissue conduit after the implantation of a Teflon tubing in a minipig at 28 days. **A** Tailored Teflon tubing with external diameter 3.9 mm, 6.0 cm in length. **B** A Teflon tubing was implanted into the subcutaneous pocket on abdomen of a minipig through a small incision. **C** The tubular template covered with a capsule of living tissue was harvested with the minimally invasive techniques. **D** After trimming excess tissue and removing the Teflon tubing, an autologous connective tissue conduit was prepared

Smooth and unmodified Teflon tubings with an external diameter of 3.9 mm was tailored into 6.0 cm long segments (Fig. 1A). They were subsequently immersed in 75% alcohol solution for 30 min. The sterilized tubings were then inserted into the subcutaneous pocket on abdomen of miniature pigs (Fig. 1B). Four weeks after subcutaneous implantation, thick and autologous tissue conduits were formed around the tubings. They were easily harvested with little adhesion. Excess tissue was carefully removed and the tubing was extracted, leaving an autologous, smooth and thick tissue conduit that can be used as vascular graft (Fig. 1C, D).

### Decellularization and heparinization analysis

**Fig. 2:**
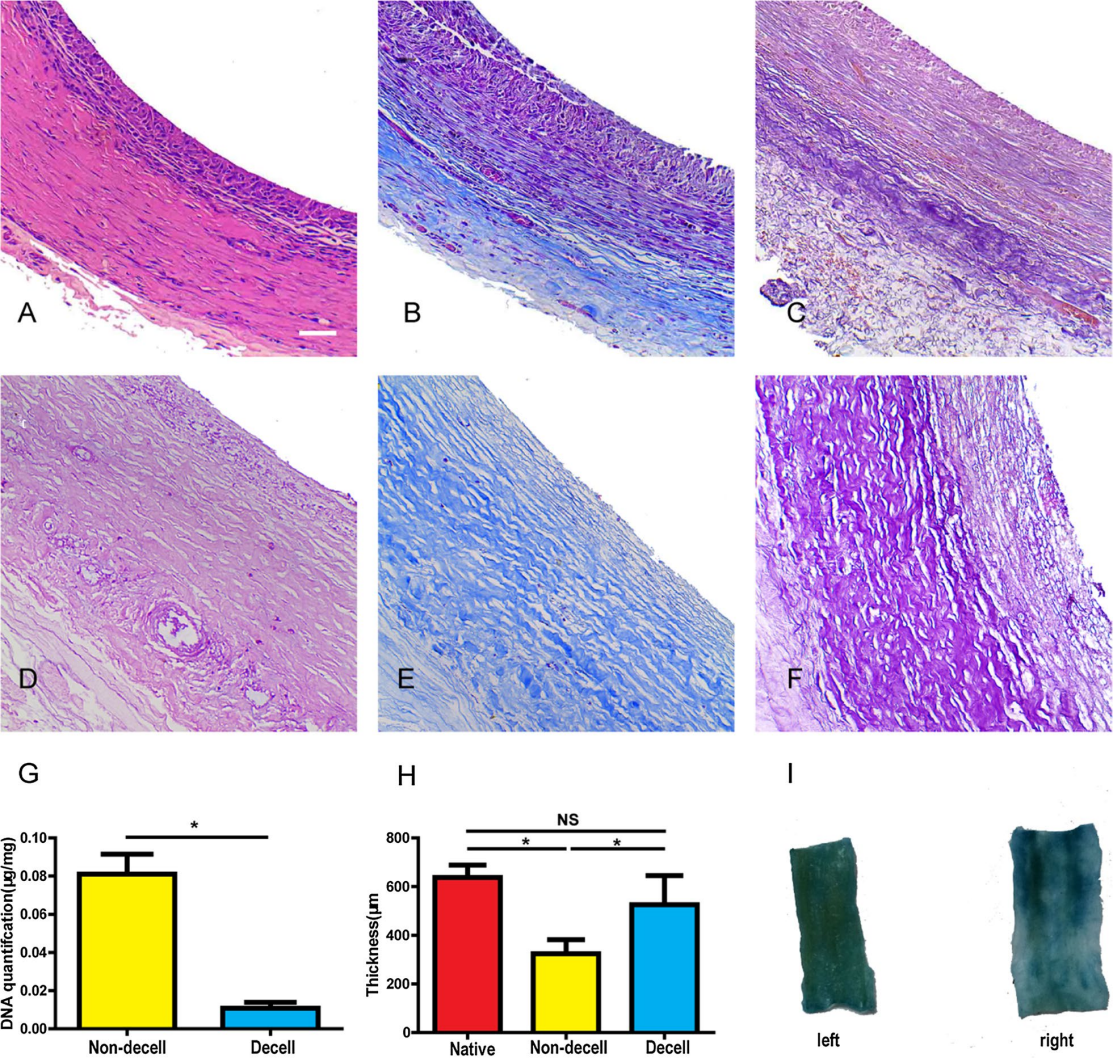
Autologous tissue conduits before (**A**–**C**) and after (**D**–**E**) decellularization. H&E staining (**A**, **D**), Masson’s trichrome staining (**B**, **E**), Verhoeff’s staining (**C**, **F**). **G** The average wall thickness of the common carotid artery, non-decellularized and decellularized autologous tissue conduits. **H** DNA quantification of non-decellularized and decellularized autologous tissue conduits. **I** Qualitative (left) and stabilization (right) test of heparin by toluidine blue for the inner surface of the tissue conduit. Scale bar, 50 μm

The tissue conduit before decellularization was composed of cells and collagen-rich extracellular matrix, the cells were supposed to fibroblast, no elastin was observed (Fig. 2A–C). After decellularization, the cellular component was seldom observed, with a well reserved collagen-rich extracellular matrix (Fig. 2D–F). DAPI staining showed the cellular component was well removed by CHAPS detergent (Data was not shown). There was a significant difference in DNA content before and after decellularization (0.011 ± 0.003 μg/mg vs 0.081 ± 0.010 μg/mg, p < 0.05) (Fig. 2G). The average thickness of the non-decellularized, decellularized and native artery were 324.1 ± 57.4 μm (n = 6), 525.7 ± 119.8 μm (n = 6), 637.4 ± 50.6 μm (n = 6) respectively, with no statistical difference in thickness between decellularized and native artery (p > 0.05) (Fig. 2H). Heparin was conjugated to decellularized tissue conduits by Sulfo-NHS/EDC. The average content of heparin that conjugated to tissue conduits was 8.2 ± 0.9 μg/mg, after a water bath at 37 °C for 12 h. The tissue conduit color was still blue stained by toluidine compared with the tissue conduit prepared by the same procedure but without PBS treatment (Fig. 2I).

### Mechanical analysis

**Fig. 3:**
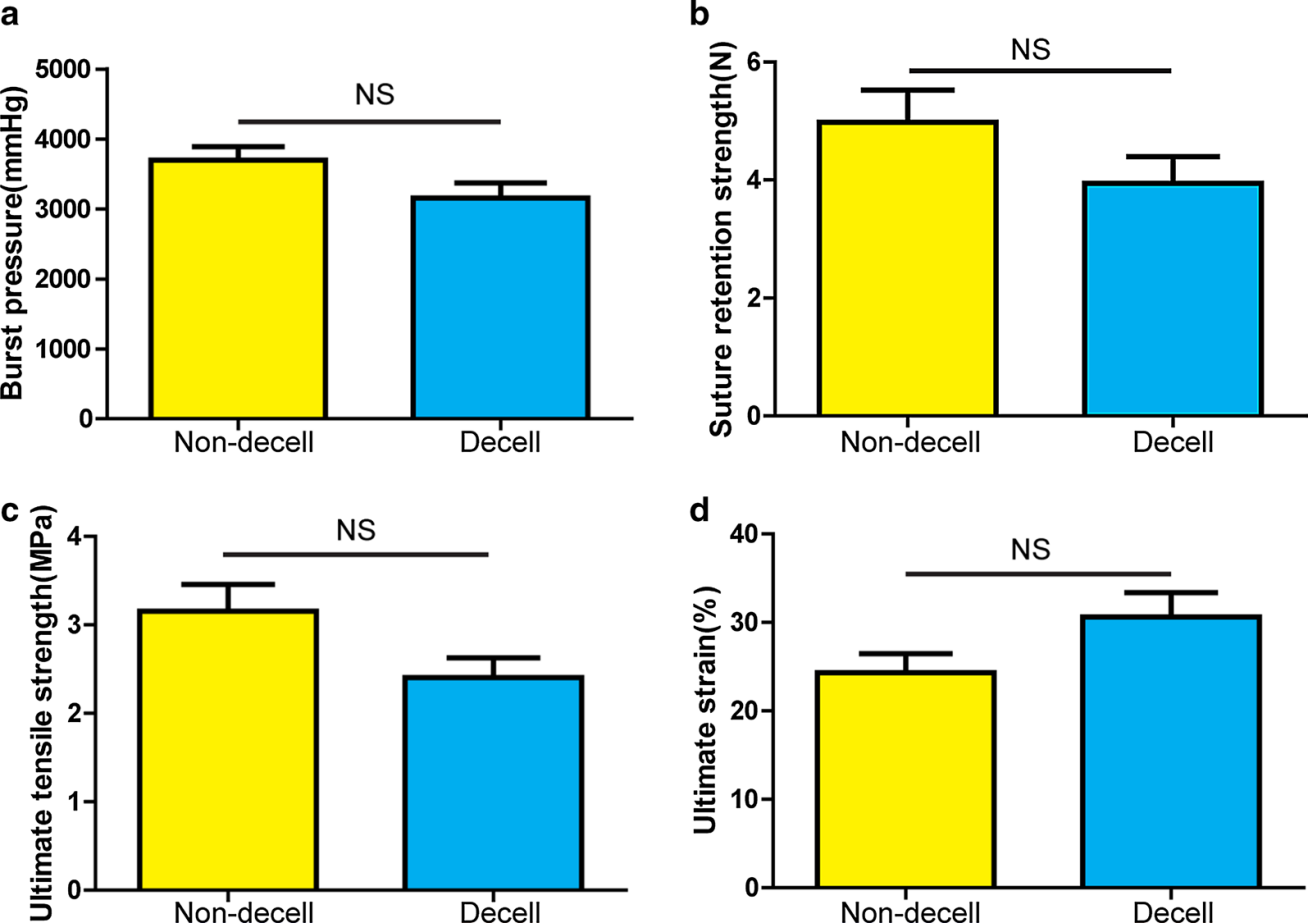
Mechanical properties of non-decellularized and decellularized autologous tissue conduits. **a** Burst pressure, **b** Suture retention strength, **c** Ultimate tensile strength, **d** Ultimate strain

Before decellularization, the burst pressure of the tissue conduit was 3696 ± 194 mmHg (n = 6), suture retention strength was 4.97 ± 0.55 N (n = 6), ultimate tensile strength was 3.16 ± 0.30 MPa (n = 6) and ultimate strain was 24.33 ± 2.15% (n = 6). After decellularization, the burst pressure of tissue conduit was decreased to 3157 ± 216 mmHg (n = 6), suture retention strength was decreased to 3.94 ± 0.46 N (n = 6), ultimate tensile strength was decreased to 2.41 ± 0.22 MPa (n = 6) while ultimate strain was increased to 30.63 ± 2.74% (n = 6). However, there were no statistically significant differences in mechanical properties between non-decellularized and decellularized tissue conduits (p > 0.05) (Fig. 3a–d).

### Surgical procedure

**Fig. 4:**
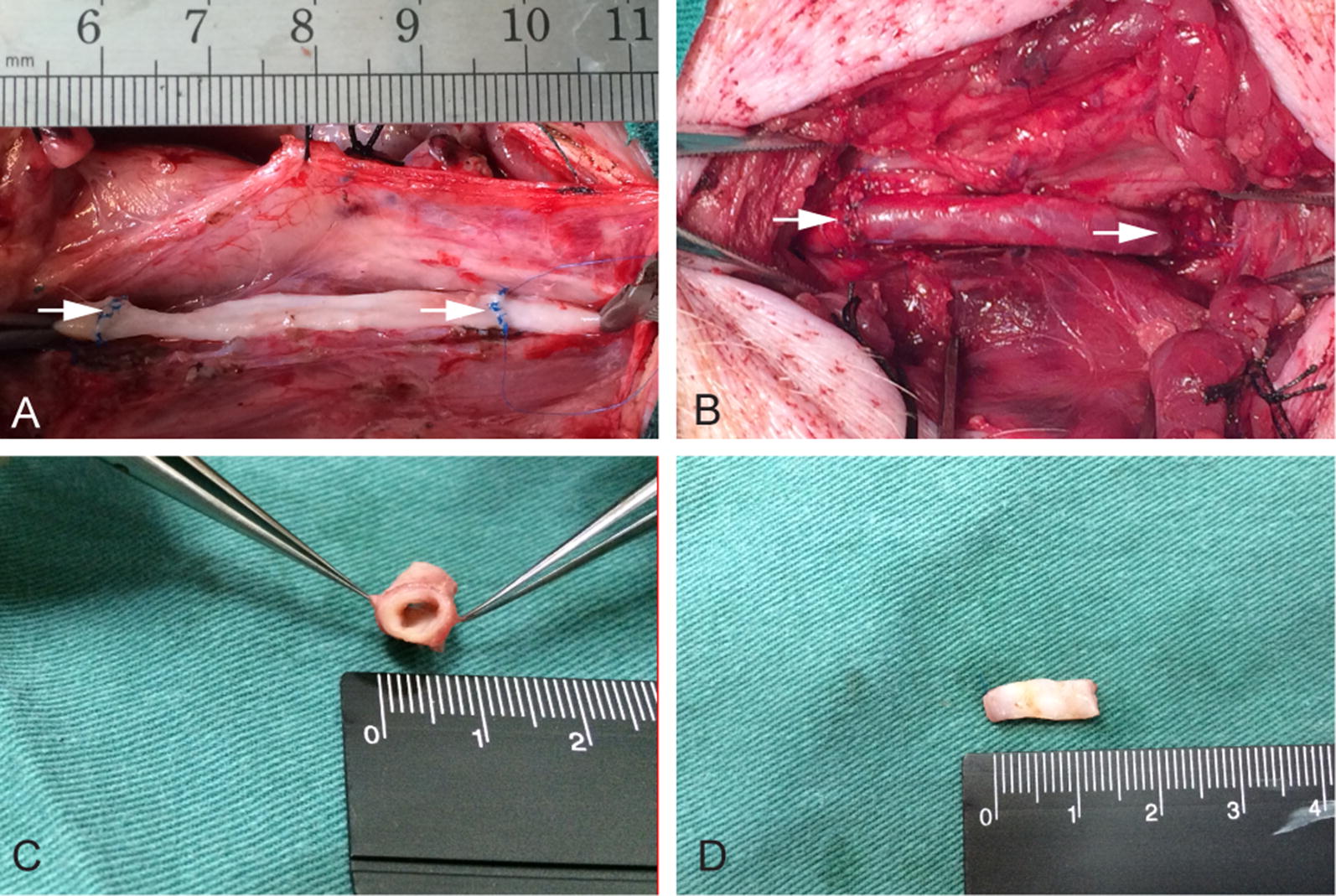
Implantation and explantation of a decellularized autologous tissue conduit conjugated with heparin as a common carotid artery interposition graft. **A** The decellularized autologous tissue conduit anastomosed as an end-to-end bypass in the porcine carotid artery. **B** Immediately post implant. White arrows indicate the anastomotic sites. **C** The sample explanted at 1 month demonstrates the patent graft. **D** Gross appearance of the inner surface of the neoartery at 2 months after implantation

The diameter of tissue conduits matched with the carotid artery diameter (Fig. 4A), it was well anastomosed to common carotid artery as an interposition graft while the blood flow in the graft was patent with obvious pulse (Fig. 4B). One and 2 months following implantation, the interposition grafts were patent with intact intima with no thrombus observed (Fig. 4C, D). 5 pigs were followed up for 1 month with the Doppler ultrasound. The patency rate was 100% (5/5) with the average inner diameter at 3.43 ± 0.05 mm (n = 5). Three pigs were followed up after 2 months with Doppler ultrasound. The patency rate was 67% (2/3) and the average inner diameter was 2.32 ± 0.14 mm (n = 2). There were no significant differences for inner diameters between follow-up grafts at 1 and 2 months.

### Histology analysis after implantation

**Fig. 5:**
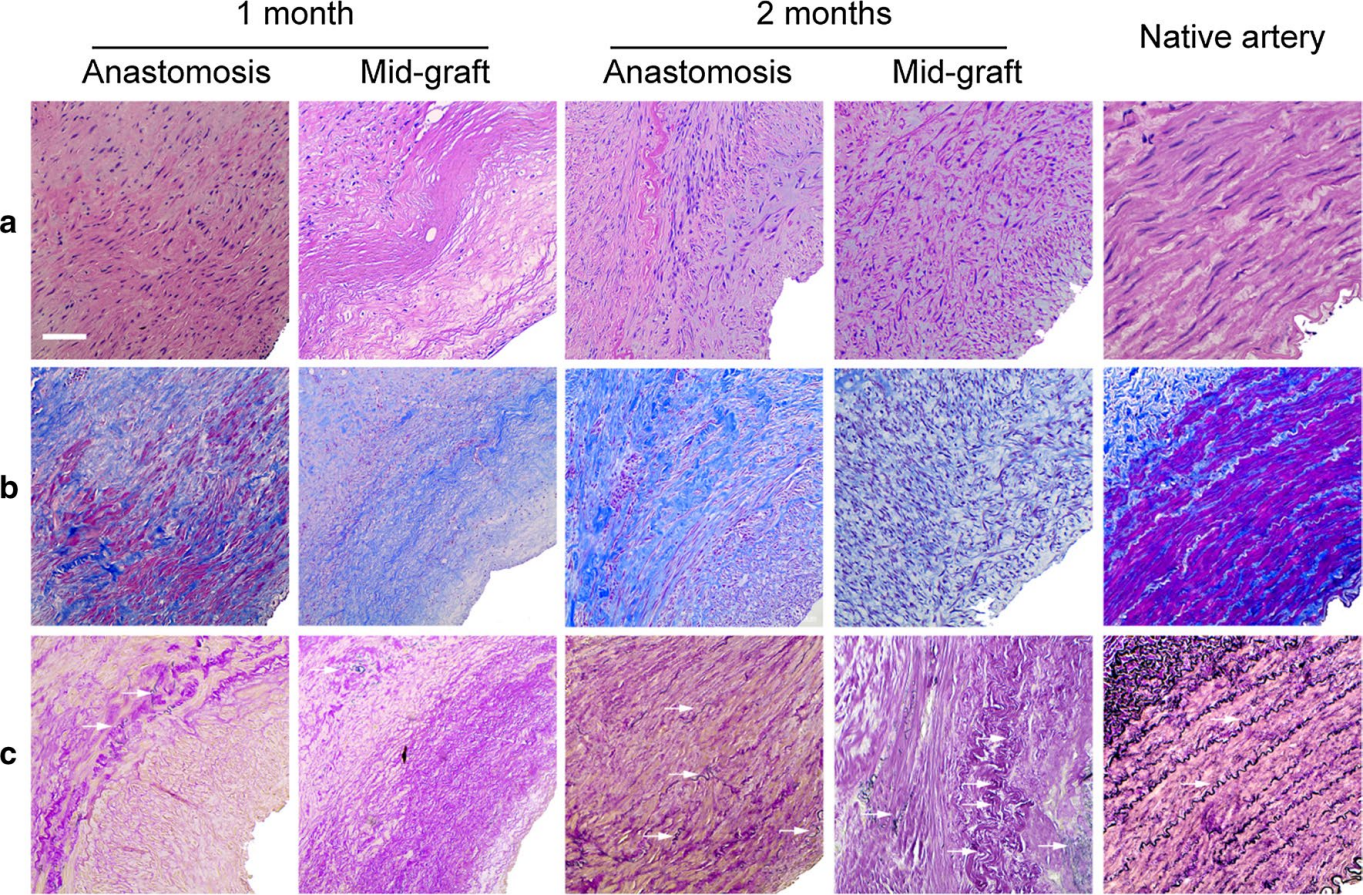
Remodeling of grafts at 1, 2 months after implantation. H&E staining (**a**) of the grafts during the transition into a neoartery. Masson’s trichrome (**b**) and Verhoeff’s staining (**c**) show collagen (blue) and elastin (black). White arrows indicate elastin. Scale bar, 50 μm

**Fig. 6:**
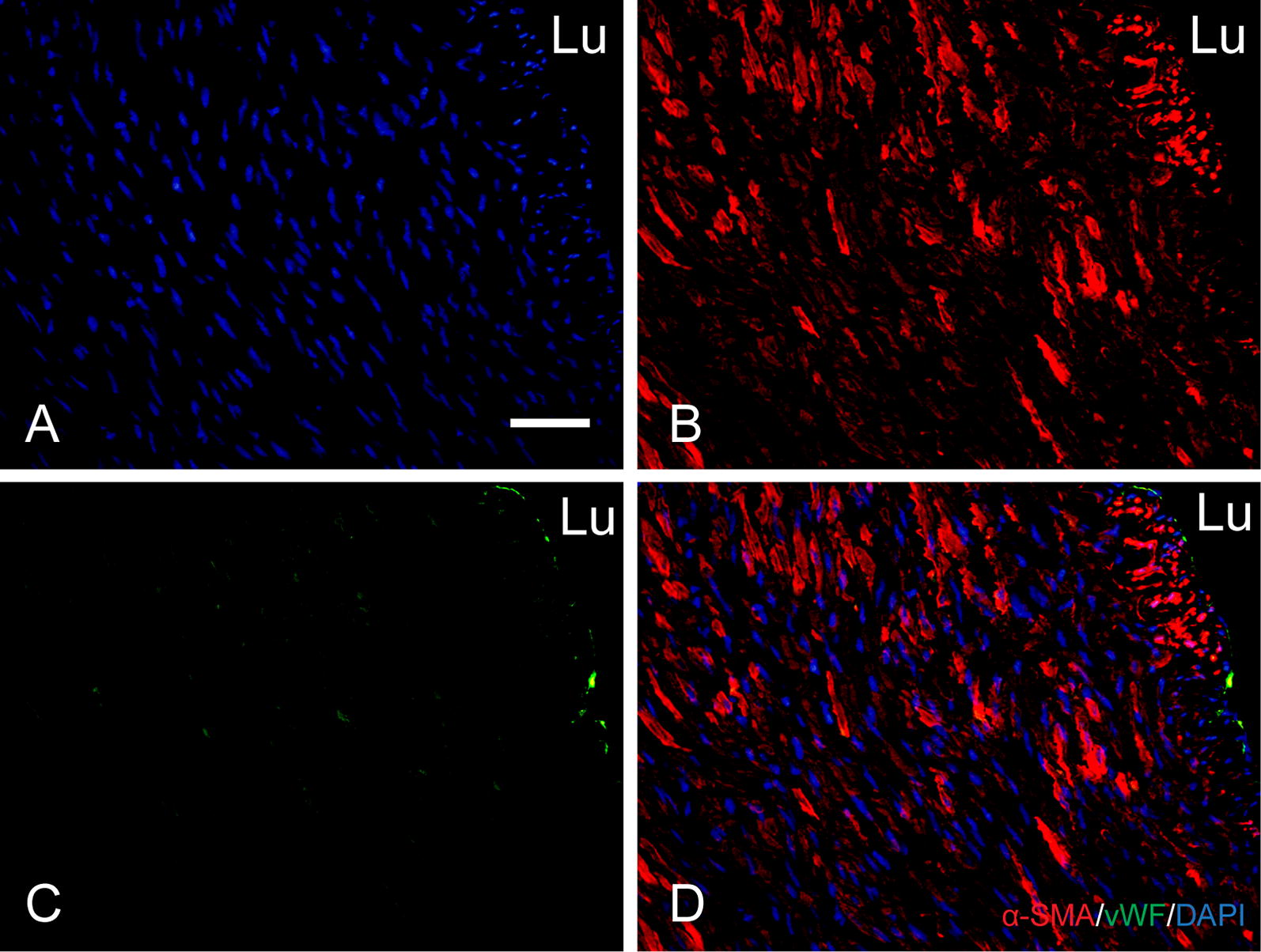
Smooth muscle cell remodeling and endothelialization of the autologous tissue conduits at 2 months after implantation. Nuclei are stained by DAPI (**A**). Double immunostaining for alpha smooth muscle actin and Von Willebrand factor (**B**–**D**). Scale bar, 50 μm. *Lu* lumen

After implantation, cells migrated into the graft wall, with few elastin formed on the graft wall at the first month after implantation, but little remodeling was observed. 2 months after implantation, there were increased number of cells that migrated into the graft walls with more mature wave-like elastin also observed in the graft wall (Fig. 5a–c). Immunofluorescence results showed that a confluent endothelial monolayer covering of the lumen, and a large number of alpha smooth muscle actin (α-SMA) positive cells were observed in the entire graft wall (Fig. 6A–D).

## Discussion

Various types of natural and synthetic scaffolds with arterial tissue cells or differentiated stem cells have recently attracted interest as potential small-diameter vascular grafts. L’heureux et al. employed tissue cells without synthetic materials to fabricate small-diameter vascular grafts which facilitated arteriovenous access in human adults for the first time [16]. They harvested autologous fibroblasts and endothelial cells from a small biopsy specimen of skin and a superficial vein. Subsequently, tissue sheets were formed by cell culture in vitro with autologous tissue engineering vascular grafts fabricated by self-assemble technology with these tissue sheets. Niklason et al. engineered vascular grafts using human allogeneic or canine smooth muscle cells grown on a tubular polyglycolic acid scaffold in a bioreactor for about 8 weeks. After the degradation of the polyglycolic acid scaffold, cellular material was removed with detergents [17]. These two types of tissue engineering vascular grafts acquired great success in animal models as well as for humans who need hemodialysis. However, the manufacturing or pre-treatment process of these grafts is complicated, expensive, and time-consuming. In this study, we demonstrated the totally autologous extracellular matrix-based grafts in small-diameter artery reconstruction, which were prepared by in vivo tissue engineering technology. It would be more suitable for clinical application because of it is totally immunologically compatible and a relatively convenient preparation process. As a candidate material for our in vivo tissue engineering, we focused our attention on its foreign body reaction in the host. For the sake of our designed objective, materials were selected that would induce only moderate foreign body reactions so that the fabricated foreign body granulomatous conduct would have appropriate thickness, mechanical properties and microstructure.

Studies have shown that, after embedded in the subcutaneous pocket of host, different materials with different surface properties would bring different tissue regeneration process [18–20]. Silastic and silicone are two materials that had been used in in vivo tissue engineering technology to fabricate small-diameter vascular grafts. They had adequate plasticity and were easily obtained, but the grafts were poor in mechanical properties [21–23]. To fabricate autologous tissue conducts with appropriate mechanical properties, one type of complex co-polymer (PEOT/PBT) was adopted in a recent study [4]. The tissue grafts had relatively excellent performance in porcine model, but the fabrication of this compound required high technology and was very time consuming. In our previous study, 5 different materials nylon, plexiglass, silicone, Teflon and polyvinyl chloride were fabricated into solid cylindrical tubes of the same size, and were then embedded in the subcutaneous pocket of rats for 4 weeks. Histology and mechanical properties of the newly formed tissue conducts were evaluated. We found that the Teflon group had the best performance in histology and mechanical properties. In our miniature pig model, the burst pressure of tissue conduits induced by subcutaneously embedding with Teflon tube was 3696 ± 194 mmHg. It was strong enough to suffer the systemic pressure of pigs and even humans, in the in vivo implantation study, no case of rupture or aneurysm formation was observed. Although properties of foreign body granulation tissue induced by different materials might be diverse, the component of this foreign body granulation tissue was relatively constant which was mainly composed of fibroblasts, small amount of macrophages, collagen I/IV and glycosaminoglycans, without elastin.

Decellularization technology is widely applied in tissue engineering. Generally, it can be achieved by physical, chemical or biologic-enzyme methods, two or three of them were always combined practically. Cells or cellular components were removed by this process, leaving the well reserved and polyporus extracellular matrix without immunogenicity and convenient for host cell migration and reconstruction. Also, decellularized tissue or organs could be preserved by lyophilization for extensive applications. CHAPS was a zwitterionic detergent used in the laboratory to solubilize biological macromolecules such as proteins. The treated artery tissue had histologically normal collagen and elastin morphology. The collagen content appeared to remain similar to that of the native artery, however, the burst pressure of arterial tissue was significantly decreased after decellularization with CHAPS alone. To improve the mechanical properties, CHAPS was used in conjunction with hypertonic detergents such as EDTA during our decellularization process. In our study, it can be seen that the cellular component was completely removed, no fracture was observed on decellularized tissue and there was a significant difference in DNA content before and after decellularization (Fig. 2G). More importantly, there were no significant difference in mechanical properties between non-decellularized and decellularized tissue conduits (Fig. 3). Theoretically, our decellularized tissue conduit was more polyporus. Therefore, it would be a more suitable microenvironment for self-renewal of the vascular architecture than a non-decellularized tissue conduit.

To improve the anticoagulation properties of the tissue engineering vascular grafts, endothelial cell seeding and covalently linked with anticoagulation substance were two ways that have been widely experimented. As mentioned before, the main component of our decellularized autologous extracellular matrix-based graft was collagen; it was one kind of protein which was found to be susceptible to thrombosis. It was proposed that the efficiency of an endothelial cell seeding was unreliable and time consuming. One study showed that the heparin-linked tissue engineering vascular graft had better anticoagulation properties than the one without heparin in vitro [24]. Another study showed that the heparin linked tissue engineering vascular graft (PTFE) had better patency rate in an animal model [25]. In our study, heparin was covalently linked to decellularized autologous tissue conduct by Sulfo-NHS/EDC. It was hypothesized that the heparin would block the collagen on our decellularized conduits, thus preventing it from being exposed to blood. Besides, the covalently linked heparin would provide an attachment point for endothelial cells. It was reported that endothelialization was complete by 1 month following grafting [26]. In order to guarantee that no thrombus would form, antiplatelet and anticoagulant drugs were employed, consequently, no thrombus was observed for at least 1 month.

Our vascular grafts used for carotid artery reconstruction in miniature pig model had a 100% patency rate at 1 month post-grafting, with no thrombus formation, neointimal hyperplasia and aneurysms observed while cells migrated into the graft wall, with only a little remodeling observed. We found that more cells migrated into the implanted graft close to anastomosis than the middle of graft. That means both circulating progenitor cells and transmural ingrowth of certain pericytes were involved in the cellularization process of artery reconstruction. To avoid complete tissue regeneration simply by transmural ingrowth of host cells, the graft length was predetermined to be 5 cm. Immunofluorescence revealed the differentiation of some vWF, α-SMA positive cells on the graft close to anastomoses. It was hard to say whether the vWF, α-SMA positive cells were differentiated from stem cells or a transmural ingrowth of mature cells. More interesting, some elastin was formed in the graft wall at the first month after implantation. This process played an important role in artery reconstruction. However, it could not be realized in a bioreactor. After 2 months, the patency rate was 67%, one pig was observed to have thrombus formation by ultrasound. After dissection, we found that the formation of the thrombus was a consequence of anastomotic stenosis. There were more α-SMA positive cells migrated into the grafts wall with a confluent endothelial monolayer covering of the lumen after 2 months. We therefore speculate that circulating progenitor cells would fall into the scaffold and induce in situ reconstruction. Meanwhile, transmural ingrowth of certain pericytes that guide vascular regeneration into the graft would occur more readily.

The limitations of our study are the lack of non-decellularized and heparin free grafts in our vasotransplantation model. We verified the feasibility of the project, with our graft showing good patency and in situ tissue regeneration, but whether decellularization and covalently linked with heparin had more benefits was not confirmed yet. From other studies, we speculate that these two processes would bring great benefits for cell migration and anti-thrombosis. To resolve these limitations, it would be necessary to implement a control group in the study for comparison.

## Conclusions

In conclusion, our decellularized autologous extracellular matrix-based vascular grafts showed good patency in a miniature pig model and good in situ cellularization and reconstruction, including relatively early endothelialization and population with smooth muscle cells and excellent mechanical property performance. Our grafts had sufficient durability and patency to be used for small-diameter artery reconstruction, particularly in peripheral arterial surgery, and might provide a wide variety of therapeutic options in cardiovascular surgery.

## Abbreviations

TEVG: tissue-engineered vascular graft
EDTA: ethylene diamine tetraacetic acid
EDC: 1-(3-dimethylaminopropyl)-3-ethylcarbodiimide hydrochloride
NHS: N-hydroxysuccinimide
MES: 2-(N-morpholino)ethanesulfonic acid
CHAPS: 3-[(3-cholamidopropyl)dimethylammonio]-1-pro-panesulfonate
DAPI: 4′,6-diamidino-2-phenylindole
